# Quantitative trait loci mapping reveals important genomic regions controlling root architecture and shoot biomass under nitrogen, phosphorus, and potassium stress in rapeseed (*Brassica napus* L.)

**DOI:** 10.3389/fpls.2022.994666

**Published:** 2022-09-12

**Authors:** Nazir Ahmad, Sani Ibrahim, Ze Tian, Lieqiong Kuang, Xinfa Wang, Hanzhong Wang, Xiaoling Dun

**Affiliations:** ^1^Key Laboratory of Biology and Genetic Improvement of Oil Crops, Oil Crops Research Institute of the Chinese Academy of Agricultural Sciences, Ministry of Agriculture, Wuhan, China; ^2^Hubei Hongshan Laboratory, Wuhan, China

**Keywords:** *Brassica napus* L., nutrient uptake, candidate genes, major QTL, QTL mapping

## Abstract

Plants rely on root systems for nutrient uptake from soils. Marker-assisted selection helps breeders to select desirable root traits for effective nutrient uptake. Here, 12 root and biomass traits were investigated at the seedling stage under low nitrogen (LN), low phosphorus (LP), and low potassium (LK) conditions, respectively, in a recombinant inbred line (RIL) population, which was generated from *Brassica napus* L. Zhongshuang11 and 4D122 with significant differences in root traits and nutrient efficiency. Significant differences for all the investigated traits were observed among RILs, with high heritabilities (0.43–0.74) and high correlations between the different treatments. Quantitative trait loci (QTL) mapping identified 57, 27, and 36 loci, explaining 4.1–10.9, 4.6–10.8, and 4.9–17.4% phenotypic variances under LN, LP, and LK, respectively. Through QTL-meta analysis, these loci were integrated into 18 significant QTL clusters. Four major QTL clusters involved 25 QTLs that could be repeatedly detected and explained more than 10% phenotypic variances, including two NPK-common and two specific QTL clusters (K and NK-specific), indicating their critical role in cooperative nutrients uptake of N, P, and K. Moreover, 264 genes within the four major QTL clusters having high expressions in roots and SNP/InDel variations between two parents were identified as potential candidate genes. Thirty-eight of them have been reported to be associated with root growth and development and/or nutrient stress tolerance. These key loci and candidate genes lay the foundation for deeper dissection of the NPK starvation response mechanisms in *B. napus*.

## Introduction

In agricultural systems, nitrogen (N), phosphorus (P), and potassium (K) are the three most important minerals that limit plant growth. A considerable amount of fertilizer has been utilized to meet N, P, and K requirements to enhance crop production. As a result, improper practices have led to serious environmental issues, low fertilizer utilization efficiency, and excessive annual energy usage (Jiao et al., 2016). For example, globally, P resources are expected to be depleted by the end of the century (Mensah et al., 2020). Therefore, it is critical to breed crop varieties that efficiently use nutrients (mainly N, P, and K) (Stahl et al., 2019). These new cultivars should provide a more cost-effective approach than relying solely on fertilizer application (Tian et al., 2016).

Roots are responsible for water absorption, nutrient uptake, and anchoring the plant in the soil and thus substantially impact crop growth and yield formation (Atkinson et al., 2019; Chen et al., 2020). Root growth regulation has been the subject of extensive research and practice to boost grain yields (Thorup-Kristensen and Kirkegaard, 2016; Calleja-Cabrera et al., 2020). Enhancing root absorption and uptake of nutrients and water is encouraged to increase agricultural output and nutrient and water usage efficiency and minimize groundwater pollution (Hatfield and Dold, 2019). Plant root systems are dynamic structures that influence overall architecture by changing root branching, root angle, and root development rates. An effective root system is critical for nutrient uptake in plants. For example, in most elite cultivars, increasing the root-to-shoot ratio facilitates the uptake of P from deep soil and promotes the growth of longer root hairs to better utilize soil spatial features in order to store nutrients in shoots (Guo and Xu, 2012; Wang et al., 2019). Therefore, optimizing root and biomass-related traits such as root length, root width, root tips, root diameter, root, and shoot biomass at the seedling stage may thus provide a feasible route for understanding early variations linked with high nutrient-uptake. Moreover, it has been determined that genetic diversity of root-related traits is necessary to increase grain yield under different nutritional conditions (Carvalho et al., 2014; Liu et al., 2017). Hence, improving nutrient uptake through useful variation in seedling root and biomass traits under different growth conditions may be a sustainable long-term strategy for developing superior cultivars (Lynch, 2019).

An effective strategy for enhancing yield production under abiotic stress conditions requires genetic assessment of quantitative traits that determine crop adaptation to adverse conditions (Li et al., 2014). It is important to note that crop performance is the consequence of thousands of gene interactions, as well as environmental and cultural practices (Collins, 2008); it is obvious that assessing quantitative trait loci (QTLs) is a powerful tool for dissecting complex quantitative traits that have been widely studied in various crops such as in wheat (Ren et al., 2017), rice (Jewel et al., 2019), maize (Sun et al., 2021), and rapeseed (Wang et al., 2017; Dun et al., 2019; Ibrahim et al., 2021; Li et al., 2021). As QTLs were identified to be linked with phenotypic variance, the corresponding loci may be amplified and thus could be used for phenotypic improvement (Liu et al., 2021; Ma et al., 2021; Peltier et al., 2021). Understanding the genetic basis for nutrient acquisition related to root development is crucial in plant breeding (Wissuwa et al., 2016). Although extensive advances have been made in understanding how plants respond to nutrition stress, many genetic bases for nutrient tolerance remain still unclear in rapeseed (Congreves et al., 2021). Only a few studies have been conducted on QTLs that allow plants to adapt to varying N, P, and K levels under uniform conditions (Guo and Xu, 2012; Wang et al., 2012a; Shen et al., 2019).

Rapeseed (*Brassica napus* L.), a globally grown *Brassica* genus crop, is an essential vegetable oil source that humans have consumed. Understanding the molecular processes influencing root development is critical for evaluating root system architecture (RSA), nutrient efficiency, and rapeseed yield potential (McGrail et al., 2020). A panel of 236 rapeseed recombinant inbred lines (RILs) was used in this study to explore root and biomass-related traits of seedling plants in hydroponics under low Nitrogen (LN), low phosphorous (LP), and low potassium (LK) conditions. The study’s ultimate objectives were as follows to (i) ascertain QTLs for root and biomass-related traits under LN, LP, and LK conditions (ii) detect major QTL clusters and determine their consistency across environments and different NPK nutritional routines to locate places with breeding prospects (iii) determine sequence variation in the crucial candidate genes (iv) find crucial candidate genes in the major QTL clusters.

## Materials and methods

### Plant materials

A cross of “Zhongshuang 11 (ZS11)” and “4D122” generated the RIL population (F_2:6_) generation used in this experiment. The F_1_ generation plants were self-pollinated to obtain F_2_ generation seeds, and the F_2_ generation was continuously selfed by the single seed descent method. F_2:6_ seeds were obtained as the recombined inbred line population, and 236 lines were randomly selected from the (F_2:6_) generation and used for this experiment (Kuang et al., 2022). The two parents were used for the pilot experiment first to find the suitable concentration of nitrogen, phosphorus and potassium, the results of which were used for low-NPK treatment for screening the RIL population.

### Experimental design and hydroponic culture condition

The two parents were grown hydroponically and analyzed in two independent trials with a completely random design at the Chinese Academy of Agricultural Sciences’ Oil Crops Research Institute in Wuhan, China. The seeds germinated in the greenhouse for 6 days, with 2 days of darkness and 4 days of light, before being transplanted into smaller blue plastic basins (34 cm × 26 cm × 12 cm) with a quarter-strength nutritional solution. A standard Hoagland’s solution (Hoagland and Arnon, 1950), with consistent concentrations of other elements, and seven nitrogen, eight phosphorus, nine potassium concentrations were used, including 15, 3.5, 1.0, 0.75, 0.5, 0.375, 0.3 mMN^+^, 1, 0.1, 0.05, 0.025, 0.01, 0.007, 0.005, 0.003 mMP^+^ and 6, 0.6, 0.3, 0.15, 0.075, 0.05, 0.025, 0.01, and 0.005 mMK^+^, respectively. The full-strength modified Hoagland’s solution contained 5 mmol L^–1^ Ca (NO_3_)_2_.4H_2_O, 5 mmol L^–1^ KNO_3_, 2 mmol L^–1^ MgSO_4_.7H_2_O, 1 mmol L^–1^ KH_2_PO_4_, 0.05 μM EDTA-Fe, 46 μM H_3_BO_3_, 14 μM MnCl_2_.4H_2_O, 0.77 μM ZnSO_4_.7H_2_O, 0.32 μM CuSO_4_, and 0.44 μM Na_2_MoO4.2H_2_O. Quarter strength, half strength, and full-strength nutritional solutions were utilized in that order for the first, second, and third weeks. Weekly, the nutrient solution was changed. Seedlings were grown under a 16/8 h light-dark cycle, a daily light intensity of 180 μmol photons m^–2^ s^–1^, a day and night temperature of 26/21°C, and relative air humidity of 50–70%.

The nutrient stress of 236 “ZS11/4D122” RILs was then evaluated in the hydroponic culture at three-macronutrient stress concentrations, low N with 0.5 mM N, low P with 0.01 mM P, and low K with 0.001 mM K, respectively, under the same concentration of other elements. Three trials were repeated three times for each stress (LN1, LN2, LN3, LP1, LP2, LP3, LK1, LK2, and LK3). Six seedlings were grown for each genotype, and the nutrient solution was changed regularly as descried for pilot experiment.

### Trait measurements

Three individual plants from each genotype were collected to conduct phenotypic identification, and each plant was divided into root and shoot sections. Five root morphology traits (RMT) viz. total root length (TRL), total root surface area (TSA), total root volume (TRV), total number of roots (TNR) were captured through images using a scanner (EPSON V700, Japan) and further analyzed by WinRHIZO software (Pro 2012b, Canada), while primary root length (PRL) was measured manually using a ruler. Seven biomass traits (BT), including root fresh weight (RFW), shoot fresh weight (SFW), were measured manually using a weighing balance. Root dry weight (RDW) and shoot dry weight (SDW) was measured after oven drying at 80°C until a constant weight was reached. Total dry weight (TDW) and total fresh weight (TFW) were estimated as SDW + RDW and SFW + RFW, respectively. The root to shoot fresh weight ratio (RSR) was calculated as the ratio between RFW and SFW.

### Phenotypic data analysis and quantitative trait loci mapping

For phenotypic and QTL analysis, all 12 investigated traits were represented by the best linear unbiased estimation (BLUE) value of three plants per genotype under NPK-stress only since the results of QTL mapping under normal conditions were shown in the previous study (Kuang et al., 2022). The “*PerformanceAnalytics*” package in R software was used to calculate Pearson correlation at a significance level of *P* < 0.05. For all traits under stress treatments, analysis of variance (ANOVA) and broad-sense heritability (*h*^2^) were performed with QRL IciMapping 4.1^1^ with the ANOVA function. A total of three phenotypic datasets (LN-BLUE, LP-BLUE, and LK-BLUE) of each trial were used to map QTLs based on the construction of a genetic linkage map of the RIL population using a *Brassica 50K SNP Chip* (Kuang et al., 2022). QTL mapping was performed with the help of the software Windows QTL Cartographer 2.5, using the composite interval mapping (CIM) approach (Wang et al., 2012b). A permutation test was performed 1,000 times with a walking speed of 1 cM at a significance level of *P* < 0.05 to reduce the type-I experimental error rate (Churchill and Doerge, 1994).

The approach suggested by Wang et al. (2016) was used for QTL integration and nomenclature. To distinguish loci identified under LN from those identified under LP and LK, “LN” was added to the QTL name. Stoll et al. (2000) defined a QTL cluster as the two markers that are closest to each other and have an overlapping confidence interval (CI). Accordingly, a QTL cluster was defined as two or more significant QTLs with overlapping CI, expressed as a map distance (LOD ≥ 2.5), and labeled “qc.”

### Mining of candidate genes and protein interaction analysis for major quantitative trait loci clusters

In accordance with Cai et al. (2015), candidate genes were identified. All SNPs in the genetic map were used to confirm the alignment of the physical and genetic maps. Illumina Inc. created 50 K probe sequences to locate homologous loci using the NCBI local blastn program against the *Darmor-bzh* reference genome for *B. napus* (Rotmistrovsky et al., 2004; Chao et al., 2017). QTL regions were defined as genomic regions aligned with QTL’s confidence interval, and genes found within the QTL were classified as candidate genes for the QTL (Chao et al., 2017). SNP/insertion-deletion (InDel) variants of potential genes were examined using the two parents’ re-sequencing data (PRJNA868428). To explore the functional interactions between the genes, we used the STRING database^2^ to build a protein interaction network using 264 potential candidate genes. We used the Arabidopsis thaliana homologous genes to find protein-protein interaction network.

## Results

### Phenotypic variations of investigated traits under NPK stress conditions

**FIGURE 1:**
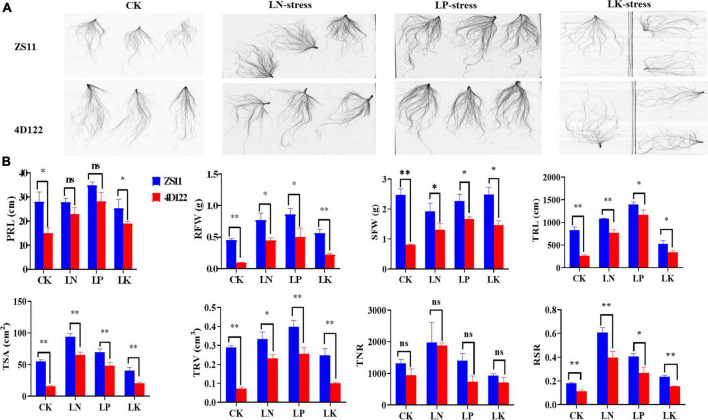
**(A)** Phenotype of the rapeseed parental lines ZS11 and 4D122 after 3 weeks under hydroponic conditions. **(B)** Comparison of parents (ZS11 and 4D122) for the investigated traits under LN/LP/LK. ^**^ and * Significant at 1 and 5% levels of probability, respectively.

A total of 12 traits, five root morphology traits (RMT) viz. PRL, TRL, TSA, TRV, TNR, and seven biomass traits (BT), RFW, SFW, RDW, SDW, TDW, TFW, and RSR, were investigated for both the parents and RIL population planted in three hydroponics trails under LN, LP, and LK treatments, respectively. Parents “ZS11 and 4D122” exhibited obvious phenotypic differences under CK/LN/LP/LK conditions (Figure 1A). The parent Zhongshuang11 (ZS11), showed significant advantages for most of the investigated traits under either CK or NPK treatment over that of another parent, 4D122 (Figure 1B). These extensive genetic variations between the two parents indicated different genetic effects on the studied traits.

**FIGURE 2:**
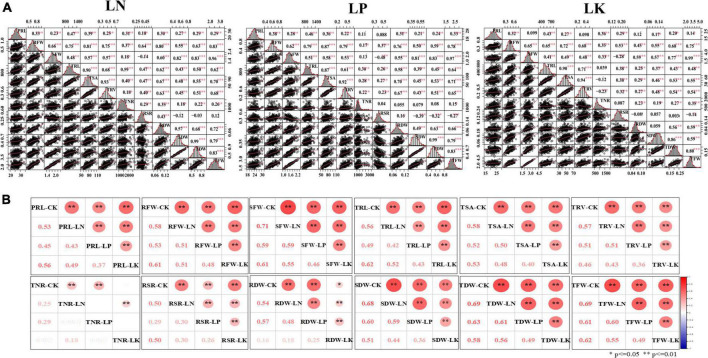
Correlation, frequency distribution and scatter plot analysis of the investigated root and biomass traits. **(A)** Correlations of captured traits under LN/LP/LK. **(B)** Correlations of each captured trait among the four treatment conditions (CK/LN/LP/LK). **, * Significant at 1 and 5% levels of probability, respectively.

ANOVA revealed that genotypes, treatments, and genotype × treatment interactions significantly affected nine traits (PRL, RFW, SFW, TRL, TSA, TRV, TNR, RSR, and TFW) (Supplementary Table 1). The phenotypic performance and broad-sense heritability *(h^2^)* for the examined traits in the RIL population are displayed (Table 1). Among the RIL population, higher and lower values than those in two parents were found under NPK-stress, implying the possibility of transgressive variation and the presence of negative and positive alleles in both parents (Table 1). The estimated *h*^2^ for the studied traits under LN/LP/LK ranged from 0.55 to 0.74, 0.43–0.73, and 0.47–0.70, respectively. High *h*^2^ values indicated that the genetic analysis of investigated traits in this study was reliable and suitable for QTL mapping. The coefficients of variation (CV) for different root and biomass traits under LN/LP/LK ranged from 10.9 to 21.3, 9.6–39.1, and 12.4–34.7%, respectively. The absolute skewness and kurtosis values for some of the investigated traits were less than 1.0 (Table 1 and Supplementary Table 2). All the studied traits exhibited normal or skewed normal distribution under LN/LP/LK (Figure 2A). These suggested that multiple genes controlled the corresponding traits.

**TABLE 1:** Descriptive statistics for investigated traits under NPK-stress in the recombinant inbred line (RIL) population.

Traits	Treat^a^	Mean	Min^b^	Max^c^	SD^d^	CV^e^ (%)	Skewness	Kurtosis	*^f^h^2^*
PRL (cm)	LN	26.2	18.7	35.1	2.85	10.9	0.33	0.23	0.58
	LP	23.8	18.2	31.7	2.28	9.6	0.34	0.52	0.49
	LK	22.6	15.0	29.9	2.81	12.4	−0.11	−0.18	0.61
RFW (g)	LN	0.687	0.451	1.074	0.11	16.2	0.56	0.55	0.70
	LP	0.629	0.418	0.893	0.09	14.2	0.43	0.41	0.61
	LK	0.474	0.268	0.835	0.08	17.6	0.38	0.97	0.70
SFW (g)	LN	1.827	1.284	2.464	0.23	12.8	0.06	−0.30	0.73
	LP	1.574	1.027	2.420	0.24	15.2	0.60	0.77	0.73
	LK	2.607	1.363	4.388	0.40	15.4	0.36	1.53	0.62
TRL (cm)	LN	1061.6	719.6	1536.0	154.60	14.6	0.56	0.21	0.65
	LP	976.3	639.0	1530.7	138.34	14.2	0.74	1.44	0.60
	LK	568.2	357.8	874.0	93.85	16.5	0.26	−0.01	0.56
TSA (cm^2^)	LN	77.7	51.0	113.7	11.17	14.4	0.40	0.16	0.63
	LP	73.1	44.4	111.9	10.58	14.5	0.64	0.71	0.58
	LK	49.2	28.6	72.5	8.42	17.1	0.08	−0.15	0.49
TRV (cm^3^)	LN	0.465	0.279	0.713	0.08	16.8	0.32	0.24	0.59
	LP	0.448	0.213	0.711	0.08	18.1	0.54	0.36	0.58
	LK	0.349	0.166	0.558	0.07	20.4	0.15	−0.10	0.47
TNR	LN	1471	816	2601	313.40	21.3	0.65	0.58	0.55
	LP	1295	688	3549	506.60	39.1	2.16	5.10	0.43
	LK	850	390	1996	295.20	34.7	1.15	1.20	0.52
RSR	LN	0.389	0.251	0.611	0.05	12.4	0.53	1.85	0.62
	LP	0.415	0.287	0.657	0.05	13.2	0.68	1.29	0.58
	LK	0.185	0.124	0.262	0.03	14.3	0.48	0.45	0.68
TFW (g)	LN	2.514	1.773	3.442	0.32	12.7	0.11	−0.26	0.74
	LP	2.202	1.490	3.288	0.30	13.7	0.57	0.95	0.72
	LK	3.081	1.700	5.224	0.46	14.9	0.34	1.86	0.63
RDW (g)	LN	0.100	0.056	0.153	0.02	15.8	0.20	0.45	–
	LP	0.086	0.055	0.138	0.01	16.1	0.40	0.48	–
	LK	0.071	0.033	0.138	0.02	21.1	1.10	2.73	–
SDW (g)	LN	0.556	0.372	0.807	0.08	14.3	0.23	0.24	–
	LP	0.456	0.328	0.683	0.07	14.7	0.48	0.06	–
	LK	0.136	0.073	0.188	0.02	17.6	−0.26	−0.37	–
TDW (g)	LN	0.656	0.442	0.951	0.09	13.7	0.23	0.31	–
	LP	0.542	0.387	0.794	0.07	13.8	0.44	0.18	–
	LK	0.208	0.113	0.283	0.03	14.0	−0.24	0.05	–

^a^Treat, treatment; ^b^Min, minimum; ^c^Max, maximum; ^d^SD, standard deviation; ^e^CV, coefficient of variation; ^f^h^2^, heritability.

### Correlation analysis among the investigated traits

Significant and strong correlations were observed among the investigated traits under LN/LP/LK conditions (Figure 2A). Consistent with the correlations under the control condition (Kuang et al., 2022), RFW revealed significant and positive correlations with the other six BT, SFW (*r* = 0.66, 0.62, and 0.65, *P* < 0.01), SDW (*r* = 0.64, 0.50, 0.55, *P* < 0.01), RDW (*r* = 0.74, 0.76, 0.45, *P* < 0.01), TDW (*r* = 0.67, 0.59, 0.68, *P* < 0.01), and TFW (*r* = 0.75, 0.78, 0.75, *P* < 0.01), as well as the four RMT, TRL (*r* = 0.60, 0.79 and 0.65, *P* < 0.01), TSA (*r* = 0.73, 0.87, 0.72, *P* < 0.01), TRV (*r* = 0.72, 0.79, 0.68., *P* < 0.01), TNR (*r* = 0.37, 0.17, 0.35, *P* < 0.01), under LN/LP/LK conditions, respectively. SFW also displayed positive and significant correlation with the four RMT, TRL (*r* = 0.30, 0.51, 0.41, *P* < 0.01), TSA (*r* = 0.44, 0.58, 0.49, *P* < 0.01), TRV (*r* = 0.50, 0.53, 0.48, *P* < 0.01), TNR (*r* = 0.28, 0.13, 0.33, *P* < 0.01). Other root and biomass traits also revealed a significant and positive correlation with each other. RSR showed significant and negative correlations with SFW, SDW, TFW, and TDW. In addition, under four different treatment conditions, CK/LN/LP/LK, significant and positive correlations were found among the studied traits (Figure 2B). For examples, correlation coefficients for PRL ranged from 0.37 to 0.56, RFW (0.48–0.61), SFW (0.46–0.71), TRL (0.42–0.62), TSA (0.40–0.58), TRV (0.36–0.57), RSR (0.26–0.50), RDW (0.16–0.57), SDW (0.36–0.68), TDW (0.49–0.69), and TFW (0.49–0.69). TNR showed a weak correlation (0.18–0.25) under different treatments. These results indicated the genetic stability of these traits across different stress conditions. Moreover, high correlations proves common genetic factors controlling the studied traits under all of the CK, LN, LP, and LK treatments.

### Quantitative trait loci mapping

#### Quantitative trait loci detected for root and biomass traits under low nitrogen-stress condition

**FIGURE 3:**
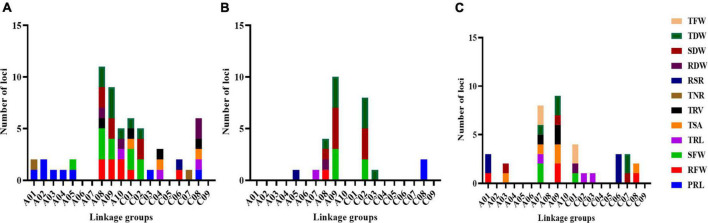
Information of loci detected for RMT and BT under LN/LP/LK conditions. **(A)** Distribution of loci on 19 linkage groups for RMT and BT under LN-stress conditions. **(B)** Distribution of loci on 19 linkage groups for RMT and BT under LP-stress conditions. **(C)** Distribution of loci on 19 linkage groups for RMT and BT under LK-stress conditions. Different colors shows the investigated traits.

Using a high-density SNP linkage mapping, a total of 57 loci associated with 12 root and biomass traits, were detected on 15 linkage groups (A01: 2, A02: 2, A03: 1, A04: 1, A05: 2, A08: 11, A09: 9, A10: 5, C01: 6, C02: 5, C03: 1, C04: 3, C06: 2, C07: 1, and C08: 6) under the LN condition in this RIL population, explaining 4.1–10.9% phenotypic variance (R^2^) (Figure 3A and Supplementary Table 3). Among these, 20 loci for RMT (including “PRL: 8, TRL: 3, TSA: 3, TRV: 4, and TNR: 2”) and 37 loci for BT (“RFW: 8, SFW: 10, RSR: 1, RDW: 4, SDW: 6, and TDW: 8”) were observed under the LN condition. High phenotypic variance (PVE) were explained by these loci for RMT and BT (“PRL: 50.6%, TRL: 18.8%, TSA: 17.9%, TRV: 30.9%, TNR: 13.1%, RFW: 45.6%, SFW: 69.9%, RDW: 23.6%, SDW: 39.1%, TDW: 54.6%, and RSR: 7.2%”). 53% (30 loci), and 47% (27 loci) were identified with positive and negative additive effects, indicating the importance of both parents toward the investigated traits.

#### Quantitative trait loci detected for root and biomass traits under low phosphorous-stress condition

A total of 27 loci were detected on seven linkage groups (A05: 1, A07: 1, A08: 4, A09: 10, C02: 8, C03: 1, and C08: 2), explaining 4.6–10.8% phenotypic variation under the LP condition (Figure 3B and Supplementary Table 4). These loci explained high PVE for different RMT and BT; i.e., PRL: 15.1%, TRL: 6.0%, RFW: 5.1%, SFW: 33.3%, RDW: 4.9%, SDW: 61.5%, TDW: 59.6%, and RSR: 5.1%. Among these, three loci for RMT (“PRL: 2, TRL: 1) and 24 loci for BT (RFW: 1, SFW: 5, RSR: 1, RDW: 1, SDW: 8, and TDW: 8”). Maximum loci (63%, 17 loci) showed a negative additive effect, indicating the contribution of parent ZS11 toward these traits.

#### Quantitative trait loci detected for root and biomass traits under low potassium-stress condition

A total of 36 loci on 10 linkage groups (A01: 3, A02: 2, A07: 8, A09: 9, C01: 4, C02: 1, C03: 1, C06: 3, C07: 3, and C08: 2) were detected under the LK-stress condition (Figure 3C and Supplementary Table 5). These detected loci explained 4.9–17.4% of the total phenotypic variation. These 36 loci explained high phenotypic variance for the investigated RMT and BT (TRL: 15.9%, TSA: 35.2%, TRV: 19.5%, RFW: 22.8%, SFW: 20.7%, RDW: 6.0%, SDW: 17.9%, TDW: 32.7%, TFW: 24.1%, and RSR: 57.0%). Among 36 loci under LK-stress condition, 11 loci for RMT (“TRL: 3, TSA: 5, and TRV: 3”), while 25 loci for BT (“RFW: 4, SFW: 3, RSR: 5, RDW: 1, SDW: 3, TDW: 5, and TFW: 4) were detected. Most detected loci showed negative effects (75%, 27 loci), consistent with those under LN and LP conditions.

#### Identification of specific and common quantitative trait loci clusters under NPK-stress

**FIGURE 4:**
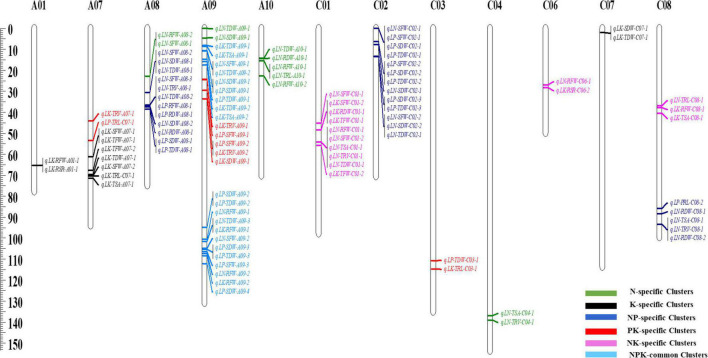
Information of quantitative trait loci (QTL) clusters in recombinant inbred line (RIL) population under LN/LP/LK conditions.

The genomic region associated with many traits is biologically intriguing because it may harbor important regulators. It signifies the existence of a single gene with a pleiotropic effect or closely linked loci controlling two or more traits (Goto et al., 2021). The shared genomic region in this study is referred to as the QTL hotspot or QTL cluster. Based on the overlapping confidence intervals, 97 out of 120 loci were integrated through QTL-meta analysis into 18 unique pleiotropic QTL clusters under NPK-stress conditions (Table 2, Figure 4, and Supplementary Table 6). These QTL clusters explained 4.6–12.8% of the total phenotypic variance. Genetic regions of these QTL clusters were detected on 12 linkage groups; A01, A07–A10, C01–04, and C06–C08. Out of 18 QTL clusters, 12 QTL clusters exhibited a negative additive effect, indicating the significant contribution of ZS11 toward the investigated traits. We then divided the detected QTL clusters into common and specific clusters (Kim et al., 2021). Based on this, we detected two NPK-common clusters, three K-specific clusters, four N-specific clusters, four PK-specific clusters, three NK-specific clusters, and two NP-specific clusters (Table 2 and Figure 4), suggesting that key loci associated with these traits under multiple environments or treatments.

**TABLE 2:** Summary of quantitative trait loci (QTL) clusters detected under NPK-stress in the recombinant inbred line (RIL) population.

QTL clusters	Type	Chr.	Traits	Treatment	Peak position (cM)	C.I^a^	Max LOD	Max R^2^ (%)^b^	Add^c^
*qc.A01-1*	*K-specific*	*A01*	RFW, RSR	LK-BLUE	65.21	61.9–67.7	8.9	12.8	(–)
*qc.A07-1*	*PK-specific*	*A07*	TRV, TRL	LK/LP-BLUE	53.41	37.0–56.2	3.8	6	(–)
*qc.A07-2*	*K-specific*	*A07*	SFW, TFW, TDW, TRL, TSA	LK-BLUE	71.31	59.6–81.4	6.2	9.2	(–)
*qc.A08-1*	*N-specific*	*A08*	RFW, SFW	LN-BLUE	22.81	17.3–24.6	4.6	6.3	(–)
*qc.A08-2*	*NP-specific*	*A08*	SFW, SDW, TDW, TRV, RFW, RDW	LN/LP-BLUE	38.61	24.9–40.5	7.2	9.4	(–)
*qc.A09-1*	*N-specific*	*A09*	TDW, SDW	LN-BLUE	4.51	0.0–8.1	5.2	7.3	(–)
*qc.A09-2*	*NPK-common*	*A09*	TDW, TSA, SFW, SDW	LN/LP//LK-BLUE	17.41	6.2–20.7	7.8	10.9	(–)
*qc.A09-3*	*PK-specific*	*A09*	TRV, SFW, SDW	LP/LK-BLUE	33.51	21.6–37.0	4.9	7.0	(–)
*qc.A09-4*	*NPK-common*	*A09*	SDW, TDW, RFW, SFW	LN/LP//LK-BLUE	112.21	89.2–117.0	8.2	10.9	(–)
*qc.A10-1*	*N-specific*	*A10*	TDW, RDW, RFW, TRL	LN-BLUE	22.71	11.5–26.5	4.2	6.5	(+)
*qc.C01-1*	*NK-specific*	*C01*	SFW, RDW, TFW, RFW, TSA, TRV, TDW	LN/LK-BLUE	55.61	44.8–60.0	5.3	7.3	(+)
*qc.C02-1*	*PK-specific*	*C02*	SFW, SDW, TDW	LP/LK-BLUE	13.41	0.0–16.6	6.2	8.5	(+)
*qc.C03-1*	*PK-specific*	*C03*	TDW, TRL	LP/LK-BLUE	114.71	101.1–127.7	3.6	5.0	(–)
*qc.C04-1*	*N-specific*	*C04*	TSA, TRV	LN-BLUE	139.01	135.9–144.7	5.8	8.0	(–)
*qc.C06-1*	*NK-specific*	*C06*	RFW, RSR	LN/LK-BLUE	28.21	24.7–30.9	8.6	11.9	(+)
*qc.C07-1*	*K-specific*	*C07*	SDW, TDW	LK-BLUE	2.01	0.0–4.2	5.0	7.6	(–)
*qc.C08-1*	*NK-specific*	*C08*	TRL, RFW, TSA	LK-BLUE	40.41	34.0–44.0	3.7	5.4	(+)
*qc.C08-2*	*NP-specific*	*C08*	PRL, RDW, TSA, TRV	LN/LP-BLUE	93.31	83.0–98.3	5.1	7.7	(+)

^a^C.I, confidence interval; ^b^R^2^, phenotypic variance; ^c^Add, additive gene effect.

Quantitative trait loci clusters that explain more than 10% of phenotypic variance (R^2^) and could be repeatedly detected under different environments or repetitions are referred to as major QTL clusters. Consequently, we detected four major QTL clusters associated with NPK-stress (Table 3 and Figure 4). Two NPK-common and two specific QTL clusters (K and NK-specific) were found among the identified major QTL clusters. Further, these QTL clusters may be used to predict candidate genes and conduct marker-assisted selection (MAS).

**TABLE 3:** Information of major quantitative trait loci (QTL) clusters with their physical position detected under NPK-stress in the recombinant inbred line (RIL) population.

Major QTL clusters	Type	Chr.	Traits	Treat	Peak position-cM (Mb)	C.I^a^	Physical position (Mb)	Max LOD	Max^b^ R^2^(%)	Add^c^
*qc.A01-1*	*K-specific*	*A01*	RFW, RSR	LK-BLUE	65.21 (15.59)	61.9–67.7	14.63–16.41	8.9	12.8	(–)
*qc.A09-2*	*NPK-common*	*A09*	TDW, TSA, SFW, SDW	LN/LP//LK-BLUE	17.41 (2.52)	6.2–20.7	0.73–3.02	7.8	10.9	(–)
*qc.A09-4*	*NPK-common*	*A09*	SDW, TDW, RFW, SFW	LN/LP//LK-BLUE	112.21 (28.42)	89.2–117.0	25.77–29.06	8.2	10.9	(–)
*qc.C06-1*	*NK-specific*	*C06*	RFW, RSR	LN/LK-BLUE	28.21 (14.64)	24.7–30.9	12.04–16.69	8.6	11.9	(+)

#### Candidate gene prediction and protein interaction analysis in the major quantitative trait loci clusters

**FIGURE 5:**
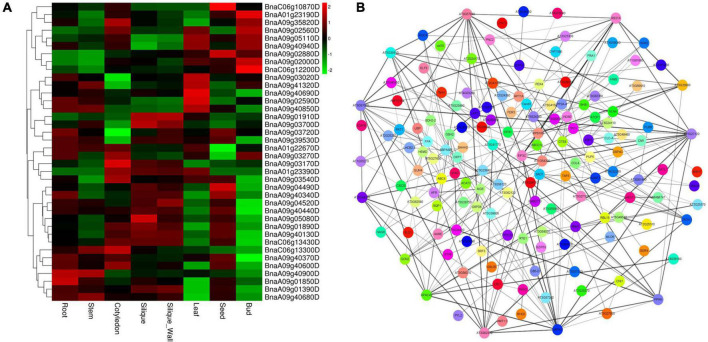
Heat map and protein interaction network analysis for the candidate genes within the major quantitative trait loci (QTL) clusters. **(A)** Expression profiles of candidate genes in eight distinct tissues; Heat map is based on the log_2_ (TPM^+1^) values. **(B)** Protein interaction network analysis.

All the annotated genes within the four major QTL clusters were retrieved according to the genome sequence of *Darmor*. As a result, 1,655 annotated gene models were found, with a gene number ranging from 168 to 631 in each major QTL cluster (Supplementary Table 7). Based on the annotation data of the retrieved genes as well as functions defined for their homologs in *A. thaliana*, the corresponding gene function was predicted (Supplementary Table 7). Furthermore, the corresponding gene information of the 1655 annotated genes in the ZS11 genome were checked. Among these, genes having high expressions in roots using the BnTIR database^3^ and having SNPs (missense variant) or indels effect (disruptive inframe deletion/insertion) between the two parents using the re-sequence data of 4D122 and the deno-genome sequence of ZS11 were selected. As a result, we found 264 potential candidate genes (Supplementary Tables 8–10) that were capable of fulfilling these two criteria. Among these, some were also found to be highly expressed in the root, stem, cotyledon, silique, silique wall, leaf, seed, and bud, implying that they are involved in plant growth and development (Figure 5A).

In living organisms, protein–protein interactions (PPIs) play a role in almost all biological processes (Rao et al., 2014); therefore, a protein interaction analysis was performed using the STRING database (Szklarczyk et al., 2021) (see footnote 2) to explore the candidate genes’ functional interactions further. Out of 264 potential candidate genes, the Arabidopsis orthologs of 262 were found that exhibit strong protein interaction with each other (Supplementary Table 10 and Figure 5B). According to previous studies, we found 38 out of 264 candidate genes, three from *qcA01-1*, 18 from *qcA09-2*, 13 and 4 from *qcA09-4*, and *qcC06-1*, known to be involved in root growth and development or nutrient utilization (Table 4). According to the protein interaction network analysis, the predicted genes were strongly interconnected and may play a major role in *NPK* stress tolerance through their interactions with other associated genes. These findings imply that these genes should be explored in greater detail to better understand their putative functions in the protein interaction network.

**TABLE 4:** Important candidate genes within the major quantitative trait loci (QTL) clusters related to root development/nutrient utilization.

Gene ID in *Darmor*	QTL cluster	Distance to Peak position (Mb)	Homologs in At.	Function annotation	References
*BnaA01g22670D*	*qc.A01-1*	0.57	*AT1G61660.1*	PFA4, enhance stress tolerance and governs the competence of pericycle cells to initiate lateral root primordium formation	Woo et al., 2012
*BnaA01g23190D*	*qc.A01-1*	0.00	*AT3G24300.1*	AMT1:3, Involved in lateral root formation and branching and ammonium homeostasis	Vega et al., 2021
*BnaA01g23390D*	*qc.A01-1*	−0.18	*AT3G24160.1*	PMP, Encodes a putative Type 1 membrane protein	Colinas and Fitzpatrick, 2016
*BnaA09g01390D*	*qc.A09-2*	1.78	*AT4G03210.1*	XTH9, Plant-type secondary cell wall biogenesis	Xu and Cai, 2019
*BnaA09g01850D*	*qc.A09-2*	1.57	*AT3G27000.1*	ARP2, Involved in cell morphogenesis	Chin et al., 2021; García-González and van Gelderen, 2021
*BnaA09g01890D*	*qc.A09-2*	1.56	*AT3G27090.1*	NRP2, involved in regulation of the protein catabolic process	Wu et al., 2022
*BnaA09g01910D*	*qc.A09-2*	1.55	*AT3G27160.1*	GHS1, Required for photosynthesis and C/N balance	Dong and Duan, 2020
*BnaA09g02000D*	*qc.A09-2*	1.52	*AT5G40650.1*	SDH2-2, Involved in mitochondrial electron transport	Samuilov et al., 2018
*BnaA09g02560D*	*qc.A09-2*	1.21	*AT5G39510.1*	VTI11, Involved in protein trafficking to lytic vacuoles	Larson et al., 2014
*BnaA09g02590D*	*qc.A09-2*	1.19	*AT3G29350.1*	AHP2, Involved in cytokinin-activated signaling pathway	Justamante et al., 2019; Islam et al., 2022
*BnaA09g02880D*	*qc.A09-2*	1.06	*AT5G48230.2*	AAT1, Sterol metabolic process	Coleto et al., 2019
*BnaA09g03020D*	*qc.A09-2*	0.98	*AT5G48430.1*	NLP7, nitrate signaling	Zhao et al., 2018
*BnaA09g03170D*	*qc.A09-2*	0.92	*AT5G48870.1*	LSM5, response to abscisic acid and root hair growth	Marzol et al., 2022
*BnaA09g03270D*	*qc.A09-2*	0.86	*AT5G49270.1*	COBL9, Involved in root epidermal cell differentiation	Canales et al., 2014; Janes et al., 2018
*BnaA09g03540D*	*qc.A09-2*	0.72	*AT5G29000.2*	PHL1, PHL1 acts redundantly with PHR1 to regulate responses to Pi starvation.	Fan et al., 2021
*BnaA09g03700D*	*qc.A09-2*	0.67	*AT5G27380.1*	GHS2, Involved in glutathione synthetases	Podg et al., 2018; Trujillo-Hernandez et al., 2020
*BnaA09g03720D*	*qc.A09-2*	0.65	*AT5G27420.1*	ATL31, Involved in Carbon/Nitrogen response for growth phase transition	Li et al., 2020
*BnaA09g04490D*	*qc.A09-2*	0.31	*AT5G25760.2*	PEX4, Involved in sucrose-dependent seedling development and reduced lateral root production	Ehrary et al., 2020
*BnaA09g04520D*	*qc.A09-2*	0.29	*AT5G25610.1*	RD22, responsive to dehydration 22 (RD22) mediated by ABA and lateral root elongation	Lee et al., 2021
*BnaA09g05080D*	*qc.A09-2*	0.02	*AT5G23950.1*	Calcium-dependent lipid-binding	Bouain et al., 2019
*BnaA09g05110D*	*qc.A09-2*	0.01	*AT5G23900.1*	RPLD13, Ribosomal protein L13e family protein	Wang et al., 2013
*BnaA09g35820D*	*qc.A09-4*	2.34	*AT3G56370.1*	IRK, Inflorescence and root apices receptor kinase	Xun and Gou, 2020
*BnaA09g39530D*	*qc.A09-4*	0.40	*AT3G61960.1*	ATG1A, Serine/threonine-protein kinase	Bedu et al., 2020
*BnaA09g40130D*	*qc.A09-4*	0.16	*AT3G62770.1*	ATG18A, Required for autophagosome formation during nutrient deprivation and senescence	Zhen et al., 2019; Huo et al., 2020
*BnaA09g40340D*	*qc.A09-4*	0.09	*AT3G62980.1*	TIR1, Encodes an auxin receptor that mediates auxin-regulated transcription	Yang et al., 2022
*BnaA09g40370D*	*qc.A09-4*	0.00	*AT3G63390.1*	Nutrients signaling	Armengaud et al., 2010
*BnaA09g40440D*	*qc.A09-4*	−0.04	*AT2G26540.1*	Encodes a uroporphyrinogen-III synthase involved in tetrapyrrole biosynthesis	Kuang et al., 2017
*BnaA09g40600D*	*qc.A09-4*	−0.11	*AT2G26300.1*	GPA1, Encodes an alpha subunit of a heterotrimeric GTP-binding protein	Sahito et al., 2020
*BnaA09g40680D*	*qc.A09-4*	−0.14	*AT2G26060.1*	CIA1, Encodes a homolog of the yeast Cytosolic Iron-sulfur protein	Lešková et al., 2020
*BnaA09g40690D*	*qc.A09-4*	−0.15	*AT2G26040.1*	PYL2, Mediate ABA-dependent regulation of protein phosphatase	Zeng et al., 2021
*BnaA09g40850D*	*qc.A09-4*	−0.20	*AT2G25570.3*	ISE3, SEL1-like repeat protein involved in plasmodesmata-mediated intercellular transport	Vijayakumar et al., 2016
*BnaA09g40900D*	*qc.A09-4*	−0.23	*AT4G32400.1*	BT1, Encodes a plastidial nucleotide uniport carrier protein required to export newly synthesized adenylates into the cytosol	Araus et al., 2016; Rossdeutsch et al., 2021
*BnaA09g40940D*	*qc.A09-4*	−0.24	*AT5G52560.1*	UPS, sugar pyrophosphorylase	Velinov et al., 2020
*BnaA09g41320D*	*qc.A09-4*	−0.48	*AT2G24540.1*	AFR, F-box protein	Rath et al., 2020
*BnaC06g10870D*	*qc.C06-1*	1.72	*AT5G51060.1*	RHD2, Involved in normal root hair elongation	Mase and Tsukagoshi, 2021
*BnaC06g12200D*	*qc.C06-1*	0.10	*AT4G30160.1*	VLN4, Encodes a major actin filament bundling protein that is involved in root hair growth	García-González and van Gelderen, 2021
*BnaC06g13300D*	*qc.C06-1*	−1.40	*AT5G40890.1*	CLC-A, Encodes a member of the voltage-dependent chloride channel	Alcock et al., 2018
*BnaC06g13430D*	*qc.C06-1*	−1.51	*AT5G41080.1*	GDPD2, Encodes a member of the glycerophosphodiester phosphodiesterase	Das et al., 2022

## Discussion

Correlation studies help breeders in identifying the fundamental traits for which selection can be based on population improvement (Jewel et al., 2019). Significant and strong positive correlations were observed among most of the studied traits under NPK-stress conditions (Figure 2A). At the same time, RSR exhibited a significant negative correlation with SFW, SDW, TDW, and TFW stress conditions. Here, we want to highlight that RSR may be a key phenotypic trait due to its sensitivity to nutritional stress; these findings are congruent with those for rapeseed (Dun et al., 2019). Highly correlated traits with a shared genetic basis were identified, demonstrating that these 12 root and biomass traits could be used to assess NPK-deficiency tolerance in RILs at the seedling stage (Liu et al., 2017). Heritability for RDW, SDW, and TDW has not been included because these dry weights were measured as sum of three plants. Root and biomass traits had significant heritabilities and genetic variations, suggesting that they might be used as primary selection criteria for optimizing nutrient use efficiency and uncovering underlying genetics (Hawkesford and Griffiths, 2019; Jewel et al., 2019). These findings confirmed prior studies by emphasizing the role of root development in improving N, P, and K efficiency under low N, P, and K conditions (Wang et al., 2017, Wang et al., 2019; Dun et al., 2019; Li et al., 2021). These kinds of variations in population for intricate root behavior may be critical for the genetic dissection of valuable loci (Danakumara et al., 2021; Sandhu et al., 2021).

Significant genetic variation among genotypes allowed researchers to investigate genetic loci linked with the observed traits (Kang et al., 2021; Roy et al., 2021). In the current study, the RIL population was phenotyped, QTL analysis was performed under LN/LP/LK treatments, and 57, 27, and 36 loci under LN, LP, and LK, respectively, that controlled nutrient deficiency were identified (Supplementary Tables 3–5). Multiple QTLs were detected for each trait, with varying contributions from both parents, revealing the possibility of epistatic interactions across both parental genomes, in which alleles from both parents act together to express these traits. This finding supports the pyramiding of QTLs for several different traits acting same time in a specific cultivar (Dormatey et al., 2020; Mei et al., 2020). The co-localization of QTLs for many traits can be attributed to the pleiotropic effect of a single gene or a network of interrelated genes, each of which affects one trait (Amoah et al., 2020; Ibrahim et al., 2021; Prakash et al., 2021). The co-localization of 18 QTL clusters in this study suggested that this genomic region could help with breeding NPK efficiency-related root and biomass traits. Among these identified QTL clusters, seven clusters (*qcA09-1, qcA09-4, qcC03-1, qcC04-1, qcC06-1, qcC08-1*, and *qcC08-2*) have been reported recently under control condition (Kuang et al., 2022). These QTLs may be in selecting a specific or common nutrient efficiency, as direct selection for one will result in indirect selection for the other. Furthermore, QTL clusters suggest that employing many QTLs to improve root and biomass traits is easier than using single QTLs (Hina et al., 2020). We found four major QTL clusters (two NPK-common and two K/NK-specific) that explained (R^2^ > 10%) among these 18 co-localized QTL clusters, demonstrating the reliability of QTL mapping. Our study’s extensive QTL cluster analysis implies that breeding programs intending to improve root and biomass traits with improved nutrient uptake efficiency should concentrate on major QTL clusters and choose loci with these regions.

Identifying potential candidate genes underlying the QTL region is extremely important for breeding programs (Wang et al., 2020; Garcia et al., 2021; Soriano et al., 2021). The sequencing and annotation of the *B. napus* genome and expression databases would contribute to the development of molecular markers and the knowledge of gene function, regulation, and expression (Lu et al., 2019). Based on gene annotations and available literature, the current experiment identified potential candidate genes influencing root and biomass traits under NPK-stress in rapeseed that underpin the four designated “QTL hotspots/major QTL clusters.” Most of these potential candidate genes were significantly expressed in root and stem tissue (Figure 5A). Many genes orthologous to *A. thaliana* were associated with root development or nutrient utilization based on functional annotation of candidate genes. For example, *BnaA01g22670D* was located at a distance of 583 Kb from the peak position of the major QTL cluster *qcA01-1* associated with root and biomass traits under LK-stress and orthologous to *AtPFA4*, which has a potential role in phosphorus starvation in *A. thaliana* (Woo et al., 2012). Another potential candidate gene (*BnaA01g23190D, AtAMT1;3*) encoding an ammonium transporter has been reported that promotes primary and lateral root growth in response to nitrate (Vega et al., 2021).

Similarly, *BnaA01g23390D* was identified at a distance of 184 Kb from the peak position of the major QTL cluster *qcA01-1*, associated with root and biomass traits under LK-stress and orthologous to *AtPMP*, which has a crucial role in nitrogen metabolism (Colinas and Fitzpatrick, 2016). In the major QTL cluster qcA09-2, some potential candidates were also detected. For example, *BnaA09g04490D* is located at a distance of 317 Kb from the peak position of *qcA09-2* and is associated with root and biomass traits under NPK-stress. An orthologous *BnaA09g04490D* (*AtPEX4*) has been reported to regulate primary nitrate response, potentially by interfering with the *TGA1* and *TGA4* transcription factors (Ehrary et al., 2020). Another gene (*BnaA09g04520D, AtRD22*) located at 296 Kb from the peak position of major QTL cluster *qcA09-2* has been demonstrated to play a key role in nitrogen use efficiency and nitrate assimilation (Lee et al., 2021). Two important genes (*BnaA09g05080D* and *BnaA09g05110D*) located at a distance of 20 and 10 Kb from the peak position of the major QTL cluster *qcA09-2*, respectively, have been reported that play a key role in root growth and development under phosphorus stress condition (Wang et al., 2013; Bouain et al., 2019). Several crucial candidate genes were also identified in the major QTL cluster *qcA09-4*. For example (*BnaA09g40130D, AtATG18A*), located at a distance of 163 Kb, has been reported to regulate nitrogen use efficiency (Zhen et al., 2019; Huo et al., 2020). Another gene (*BnaA09g40340D, AtTIR1*) found at a distance of 92 Kb from the peak position and has been reported recently that regulate root growth and thus can enhance crop production (Yang et al., 2022). *BnaA09g40370D* encoding a hypothetical protein and has been reported to regulate root growth and development in response to potassium deficiency (Armengaud et al., 2010). Another gene (*BnaA09g40440D*) located at 40 Kb upstream from the peak position of the major QTL cluster *qcA09-4* associated with root and biomass traits and has been reported that regulation nitrogen assimilation and utilization (Kuang et al., 2017). Similarly, some important candidate genes were identified in the major QTL cluster *qcC06-1*. For example, a gene (*BnaC06g12200D, AtVLN4)* has been detected at a distance of 102 Kb from the peak position and reported to regulate root growth and development under different abiotic stresses (García-González and van Gelderen, 2021). These potential candidate genes were found in strong interaction with other genes in the protein interaction network (Figure 5B). As discussed in Supplementary Table 10, these interacting genes have a potential role in root growth and development, hormone signaling pathways, and nutrient utilization. Finally, we hypothesized that the genes that were found to be orthologous to nutrient stress/tolerance genes in *A. thaliana* might be highly related to nutrient stress/tolerance in *B. napus*. Further research and validation of these genes may be carried out to confirm their role in nutrient stress/tolerance in *B. napus*. These key loci and candidate genes lay the foundation for deeper dissection of the NPK starvation response mechanisms in *B. napus.*

## Conclusion

Rapeseed oil is not only widely consumed in the human diet but also the world’s second-leading source of biodiesel. In many plant-breeding programs, developing crop varieties with stronger RSA is viewed as a way to reduce the use of NPK fertilizers by enhancing nutrient use efficiency and thereby increasing yield productivity. In the current study, the recombinant inbred line population originated from ZS11, the donor parent, and 4D122, the recipient parent, enabled us to uncover a large number of loci (120 QTLs) associated with root and biomass traits under NPK-deficiency. Among them, we detected 97 loci for different root and biomass traits that were integrated into 18 QTL clusters (NPK-specific and NPK-common). Four identified QTL clusters were further classified as major QTL clusters, comprised of several loci associated with different root and biomass architectural traits under NPK-deficiency conditions. Two of these major QTL clusters were expressed in all three stress conditions, indicating an underlying uniform basis of genetic mechanisms, contributing to the tolerance of these traits. The list of detected loci and refined clusters will facilitate further validation in systematic breeding for specific adaptability under low-input conditions and suggest that the genomic regions could be used as targets to understand the RSA mechanism better and improve nutrient use efficiency in rapeseed. In the future, the detected promising harbor QTLs will lead to the fine-mapping and molecular cloning of key loci that can be used to improve grain yield and quality under low-input fertilizer management conditions.

## Data availability statement

The data presented in the study are deposited in the NCBI database, https://www.ncbi.nlm.nih.gov/bioproject/PRJNA868428. The data is downloadable at this link: https://sra-download.ncbi.nlm.nih.gov/traces/sra74/SRR/020517/SRR21010126. Also accessible through this link: https://trace.ncbi.nlm.nih.gov/Traces/index.html?view=run_browser&acc=SRR21010126&display=data-access.

## Author contributions

XD planned and supervised the research. NA, SI, ZT, and LK performed root traits investigation and analyzed the data. NA wrote the manuscript. XD, XW, and HW contributed to modify the manuscript. All authors contributed to the article and approved the submitted version.

## Footnotes

1
https://isbreedingen.caas.cn/software/qtllcimapping/294607.htm

2
https://string-db.org/cgi/

3
http://yanglab.hzau.edu.cn/BnTIR
